# Managing a Patient Presenting With Chest Pain and Gastrointestinal Complaints Using Dupilumab

**DOI:** 10.7759/cureus.46478

**Published:** 2023-10-04

**Authors:** Kayla L Alaimo, Darren N Ramcharan, Oliver G Shaw

**Affiliations:** 1 Medicine, Saint James School of Medicine Anguilla, The Quarter, AIA; 2 Medicine, Saint James School of Medicine, Chicago, USA; 3 Emergency Medicine, Niagara Health/McMaster University, St. Catharines, CAN

**Keywords:** hypersensitivity, gastritis, chest pain, atopic dermatitis, adverse drug event, adverse drug reaction, dupilumab

## Abstract

Adverse drug reactions or adverse drug events account for a significant proportion of emergency department visits among children and adolescents. Unfortunately, rare reactions to medications may go unnoticed by clinicians due to a lack of reporting to drug surveillance and monitoring programs. We present the case of an 18-year-old male who visited the emergency department on two separate occasions after receiving dupilumab injections for his atopic dermatitis. Ten days prior to his presentation, he was evaluated in the emergency room for the onset of chest pain, five days following his first dupilumab injection. Investigations in the interim revealed no cardiac pathology. He presented with a complaint of severe abdominal pain associated with nausea and vomiting several hours after receiving his second dupilumab injection. Investigations for causes of acute gastrointestinal or anaphylactic reactions only revealed mild leukocytosis and hypokalemia. A definitive diagnosis of hypersensitivity reaction, such as anaphylaxis or serum-sickness-like reaction, could not be made at either emergency visit due to the lack of objective findings and few similar reported cases. However, the timing of each event made an adverse reaction highly suspicious as the inciting factor of this patient’s symptoms. He received oral potassium, ketorolac, and ondansetron for headache and ongoing nausea respectively. He was discharged home within a few hours after his symptoms had resolved. The limited reports and evidence of these symptoms being associated with dupilumab injections made it difficult to reach a definitive diagnosis. However, a holistic review of the patient’s history, medication list, and contextual factors revealed that a rare adverse drug reaction was a possible inciting factor on each separate occasion. Further research is required to determine the frequency and explore the existence of any causal relationship between dupilumab treatment and chest pain or gastritis in adolescent populations.

## Introduction

The World Health Organization defines an adverse drug reaction (ADR) also commonly referred to as an adverse drug event (ADE) as “a response to a drug that is noxious and unintended and occurs at doses normally used in man for the prophylaxis, diagnosis, or therapy of disease, or for modification of physiological function” [1]. ADEs are a significant cause of emergency department (ED) encounters, accounting for up to 3.5% of annual visits in some studies [2]. Across all age groups, side effects were the most common ADR chief complaint made by patients during ED visits while allergies and hypersensitivity reactions were particularly common among children and adolescents [2]. Less than 10% of these presentations typically require hospitalization or extended observation. Antimicrobial drugs, analgesic medications, and respiratory medications were found to be the most common offenders [3].

Dupilumab (trade name Dupixent) is a human monoclonal antibody indicated for the treatment of inflammatory conditions such as asthma and atopic dermatitis (eczema). This is achieved by blocking the signaling pathways of interleukin-4 (IL-4) and interleukin-13 (IL-13) in order to suppress type-2 T-helper cell (Th2) mediated reactions [4]. Drug monitoring of dupilumab users revealed that skin- and eye-related adverse events presented most commonly (32% and 21%, respectively), while infections (8%), musculoskeletal disorders (5%), blood and lymphatic disorders (3%), and immune disorders including serum sickness (3%) were less commonly reported [5]. Rare side effects of dupilumab may be related to blood vessel inflammation and can present with rash, chest pain, paresthesias, persistent fever, or shortness of breath in patients being treated for asthma [6]. Two types of classifications may be used to describe ADRs. Type A is dose-dependent reactions expected based on the pharmacology of the drug in question, and type B represents unpredictable reactions usually bizarre and idiosyncratic in nature [7]. Here, we present a case of an 18-year-old male who presented to the ED on two separate occasions shortly after receiving dupilumab injections.

## Case presentation

Chief complaint

An 18-year-old African American male presented to the ED with complaints of severe constant generalized abdominal pain for one hour.

History of present illness

The patient reported one episode of protracted non-bilious emesis with streaks of blood that occurred prior to his arrival in the ED. A second episode of non-bilious emesis occurred shortly after arrival to the ED with a resolution of bloody output. He complained of associated nausea, lightheadedness, and headache. He denied diarrhea, constipation, bloody stools, melena, fever, recent travel, and known sick contacts. This episode occurred a few hours after the patient received a 300mg injection of dupilumab.

**Figure 1:**
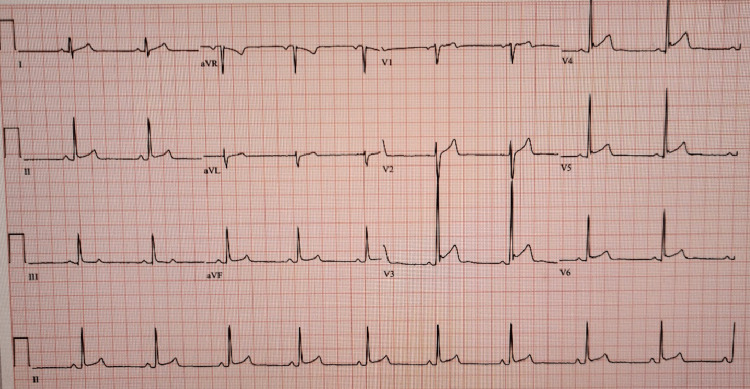
12-lead ECG showing evidence of possible early repolarization vs. pericarditis.

Ten days prior to this encounter, he presented to the ED with complaints of sharp left-sided chest pain associated with shortness of breath, five days after receiving his initial dupilumab injection (600mg). His clinical course at that time was remarkable for an abnormal electrocardiogram (ECG) with findings of diffuse ST segment elevations indicating possible early repolarization or pericarditis (Figure 1). Albuterol and ipratropium nebulizer treatments were given in the ED and upon rapid symptom improvement and a short period of observation, he was discharged home with recommendations to follow up with his primary care physician (PCP), cardiologist, and dermatologist. A cardiology assessment three days later revealed a normal high-sensitivity troponin and unremarkable findings on the echocardiogram. It was noted the patient had similar ECG findings in 2021 as observed in the ED. During a follow-up visit the following day, his dermatologist recommended he consult with allergy/immunology services.

Medical history

The patient’s past medical history consists of attention deficit hyperactivity disorder (ADHD), allergic rhinitis, atopic dermatitis, asthma, anxiety, and depression. He has known allergies to macadamia nut oil, rice, and watermelon. His past surgical history is noncontributory, and he does not have any history of frequent or similar ED visits or hospitalizations. Family history is significant for a cerebrovascular accident in his maternal grandmother at the age of 40. The patient reports never using tobacco, alcohol, or illicit substances during his lifetime or at the time of presentation to the ED on either occasion.

Medications

At the time of the encounter, the patient’s only medication was dupilumab injections every other week. It is worth noting that the patient’s records indicated he was prescribed triamcinolone 0.1% ointment, Protopic (tacrolimus) 0.1%, alclometasone 0.05% cream, Vtama (tapinarof) 1% cream, and Opzelura (ruxolitinib) 1.5% cream. However, he denied the use of any of these medications at the time of the encounter.

Clinical course and management

Based on this patient's demographics, presentation, and past medical history, we considered acute appendicitis, acute gastroenteritis, mesenteric ischemia, bowel obstruction, pancreatitis, and an acute ADR [8]. Although serum sickness-like reaction (SSLR) and anaphylaxis may be triggered by medications, this patient lacked the signs and symptoms typical of these reactions like worsening rash, arthralgia, and difficulty breathing [9,10]. Thus, we pursued other possible causes of acute abdominal pain [8].

His vital signs at the time of presentation were within normal limits and remained unchanged during this episode. Physical exam findings revealed mild abdominal tenderness, equal in all quadrants with no rebound tenderness, involuntary guarding, or abdominal wall rigidity. The patient also had several areas of healing eczematous plaques on his face, torso, and limbs. He reported these had significantly improved since he began dupilumab injections and has not since noticed a new or worsening rash. Clinically significant results from the complete blood count (CBC), complete metabolic panel (CMP), and urinalysis are summarized in Table 1. A computer-tomography (CT) scan of the abdomen and pelvis with intravenous (IV) contrast revealed no evidence of acute appendicitis, gastroenteritis, or mesenteric ischemia.

**Table 1:** Significant laboratory findings summary.

Test	Value	Reference range
White blood cell count	15.6× 10^9^cells/L	4.5 to 11.0× 10^9^cells/L
Absolute neutrophil count	13,700 cells/µL	2,500 - 6,000 cells/µL
Neutrophil %	87.7%	40%-60%
Potassium	3.2mEq/L	3.5-5.2mEq/L

The patient received 15mg of IV ketorolac for ongoing headaches, 4mg of ondansetron for nausea, and 40mEq of oral potassium chloride to replenish potassium while in the ED. His symptoms improved significantly within a few hours. He was discharged home with strict follow-up recommendations if his symptoms returned or he experienced a new onset of rash, fever, or other complaints. The patient was instructed to follow up with his PCP and dermatologist regarding the continuation of dupilumab injections.

Outcomes

Based on our findings during this encounter we could not determine a definitive diagnosis but recognized that the timing of this patient’s ED visits relative to the administration of dupilumab injections raised suspicion that an ADR was a likely etiology of this patient's symptoms. As these two acute non-specific possible ADRs had no sequelae of concern, the patient continued to receive dupilumab injections and has not experienced any similar reactions, other side effects, or complications. The patient is currently awaiting consultation with an allergist.

## Discussion

A review of both clinical trial reports and real-world monitoring reveals both gastrointestinal (GI) and cardiac side effects are uncommon in patients receiving dupilumab for atopic dermatitis, the latter being extremely rare [11,12]. Results of SINUS-24 and SINUS-52 showed that gastritis was seen in 2% of patients taking dupilumab for chronic rhinosinusitis with nasal polyps and chest pain reported in clinical trials was related to musculoskeletal etiologies and not primary cardiac conditions [6]. Because the patient’s symptoms resolved shortly with supportive care and he did not have long-lasting sequelae of these episodes, more invasive cardiac or GI workup was not pursued in the ED or during follow-up care in between each episode. The relatively benign nature of the patient's presentation and assessment on both occasions did not necessitate invasive and extensive testing such as a biopsy of the heart, blood vessels, or skin. In addition, the lack of recurrent symptoms despite subsequent dupilumab treatments obviated the indication to test for immunologic markers of vasculitis, which has been described in two previous cases of dupilumab-associated vasculitis [13]. Upon his dermatologists’ recommendation, he continued to receive dupilumab injections as the potential clinical and psychosocial benefit of reducing his atopic dermatitis was evident and the patient had a marked improvement in his eczematous plaques since receiving his initial dupilumab dose [14].

The patient information material provided by Dupixent manufacturer warns that uncommon GI side effects such as nausea and vomiting may occur. It also states chest pain may be a symptom related to the rare occurrence of drug-induced vasculitis [6]. Despite these warnings no similar cases as our patients have been noted in the literature currently. The scarcity of reported GI and cardiac side effects related to adolescent patients being treated with dupilumab added uncertainty to the degree to which we could attribute this patient’s symptoms to his injections [15]. This may be due to the rarity of such occurrences or a lack of reporting ADRs to existing drug monitoring programs, such as the Adverse Event Reporting System (AERS), which the Food and Drug Administration (FDA) claims only receives reports for 1%-10% of all ADRs [16]. In addition, chronic, insidious, and rare drug reactions often go unnoticed by physicians and therefore are unreported, with only 5% of ADRs estimated to be reported in practice [7].

## Conclusions

It is imperative to consider ADRs in the evaluation of any patient presenting with acute symptoms after the initiation or administration of novel medications. Accounting for historical and contextual factors of a patient presentation such as this one may reveal the occurrence of uncommon or rare side effects. We present this case to inform others that dupilumab is a possible cause of chest pain in patients receiving treatment for atopic dermatitis. Patients in the ED should be evaluated using the standards of care based on their chief complaint, demographics, and presentation, but clinicians should consider rare ADRs in lieu of evidence supporting other favorable diagnoses. Further investigations would be necessary to assess for any correlation between chest pain or gastritis and dupilumab injections, as well as the pathophysiologic mechanism by which this may occur. In the meantime, healthcare providers should be encouraged to describe the symptoms, treatments, and course of action that should be taken in the event an adverse reaction is suspected by their patients. More importantly, consistent reporting of suspected adverse events to drug surveillance and monitoring programs can provide clinicians with the information to easily identify and treat these patients.
